# Activity in the primary somatosensory cortex induced by reflexological stimulation is unaffected by pseudo-information: a functional magnetic resonance imaging study

**DOI:** 10.1186/1472-6882-13-114

**Published:** 2013-05-27

**Authors:** Naoki Miura, Yuko Akitsuki, Atsushi Sekiguchi, Ryuta Kawashima

**Affiliations:** 1 Department of Information and Communication Engineering, Faculty of Engineering, Tohoku Institute of Technology, Yagiyama kasumicho 35-1 Taihaku-ku, Sendai 982-8577, Japan; 2 Department of Functional Brain Imaging, Institute of Development, Aging and Cancer (IDAC), Tohoku University, Seiryo-machi 4-1, Aoba-ku, Sendai 980-8575, Japan; 3 Smart Ageing International Research Center, Institute of Development, Aging and Cancer (IDAC), Tohoku University, Seiryo-machi 4-1, Aoba-ku, Sendai, 980-8575, Japan; 4 Division of Medical Neuroimage Analysis, Department of Community Medical Supports, Tohoku Medical Megabank Organization, Tohoku University, Seiryo-machi 4-1, Aoba-ku, Sendai, 980-8575, Japan; 5 Division of Developmental Cognitive Neuroscience, Institute of Development, Aging and Cancer (IDAC), Tohoku University, Seiryo-machi 4-1, Aoba-ku, Sendai, 980-8575, Japan

**Keywords:** Reflexology, Neuroimaging, Functional magnetic resonance imaging, Primary somatosensory cortex, Placebo effect

## Abstract

**Background:**

Reflexology is an alternative medical practice that produces beneficial effects by applying pressure to specific reflex areas. Our previous study suggested that reflexological stimulation induced cortical activation in somatosensory cortex corresponding to the stimulated reflex area; however, we could not rule out the possibility of a placebo effect resulting from instructions given during the experimental task. We used functional magnetic resonance imaging (fMRI) to investigate how reflexological stimulation of the reflex area is processed in the primary somatosensory cortex when correct and pseudo-information about the reflex area is provided. Furthermore, the laterality of activation to the reflexological stimulation was investigated.

**Methods:**

Thirty-two healthy Japanese volunteers participated. The experiment followed a double-blind design. Half of the subjects received correct information, that the base of the second toe was the eye reflex area, and pseudo-information, that the base of the third toe was the shoulder reflex area. The other half of the subjects received the opposite information. fMRI time series data were acquired during reflexological stimulation to both feet. The experimenter stimulated each reflex area in accordance with an auditory cue. The fMRI data were analyzed using a conventional two-stage approach. The hemodynamic responses produced by the stimulation of each reflex area were assessed using a general linear model on an intra-subject basis, and a two-way repeated-measures analysis of variance was performed on an intersubject basis to determine the effect of reflex area laterality and information accuracy.

**Results:**

Our results indicated that stimulation of the eye reflex area in either foot induced activity in the left middle postcentral gyrus, the area to which tactile sensation to the face projects, as well as in the postcentral gyrus contralateral foot representation area. This activity was not affected by pseudo information. The results also indicate that the relationship between the reflex area and the projection to the primary somatosensory cortex has a lateral pattern that differs from that of the actual somatotopical representation of the body.

**Conclusion:**

These findings suggest that a robust relationship exists between neural processing of somatosensory percepts for reflexological stimulation and the tactile sensation of a specific reflex area.

## Background

Reflexology is an alternative medical practice that produces beneficial effects on the human body by applying pressure to specific points or areas on the feet, hands, and ears called “reflex areas.” It is a kind of massage therapy that is performed without using any special instruments (i.e., uses hands only). The modern concept of reflexology was introduced in both the U.S. and Europe in the early 20th century [1], and it is believed to help eliminate stress, improve blood circulation, and restore the psychological balance of the body [2]. Although it is practiced throughout the world and several clinical studies have shown positive effects of this practice, the precise mechanisms underpinning its clinical effectiveness have not been elucidated [3-5]. Furthermore, the physiological basis of reflexology is not fully understood. Each reflex area is believed to correspond to an organ or part of the human body; however, this correspondence had not been sufficiently investigated. Recently, neuroscientific research has examined the scientific underpinnings of complementary and alternative medicine such as acupuncture. Goldmann et al. [6] found that adenosine metabolism may influence the local antinociceptive effect of acupuncture, and Imai et al. [7] reported with acupuncture an improvement in autonomic function imbalance in rats under restraint stress. Previous neuroimaging studies using acupuncture have reported that acupoints related to different functions activate different areas of the primary somatosensory cortex (SI) [8] or different patterns of neural activity in the frontal–limbic–striatal regions [9]. Yoo et al. reported a difference in neural activity between real and sham acupunctural stimulation in the primary and secondary somatosensory cortex [10]. As for touch massage, including reflexology, a relaxing effect of reflexology was observed using electroencephalography (EEG) [11], pleasant touch massage induced activation of the pregenual anterior cingulate cortex [12], and reflexological treatments affected resting-state neural activity in the retrosplenial/posterior cingulated cortex [13].

In our previous study, we sought to provide experimental evidence of a somatotopical relationship between cortical activity in SI and sensory stimulation of reflex areas on the foot using functional magnetic resonance imaging (fMRI) [14]. We found that the area activated by stimulation of the reflex area was consistent with the somatotopic representation of the corresponding or a neighboring body part in SI. We interpreted these results as evidence of a physiological relationship between the reflex area and a body part or organ. However, an alternative interpretation is that the findings were the result of the placebo effect; it is possible that a sensory expectation resulting from *a priori* knowledge of the reflex area influenced the cognitive processing of the perceived reflexological stimulation. Blankenburg [15] reported that a somatosensory illusion called the “cutaneous rabbit” [16] evoked cortical activity in SI that somatotopically corresponded to the illusory percepts. Furthermore, this illusion has been shown to occur outside the body using a stick held across the left and right index fingers [17], indicating top-down cognitive processing such as might be observed in a body–object interaction when processing a tactile sensation. Thus, it is necessary to determine whether top-down modulation of somatosensory percepts resulting from *a priori* knowledge of the reflex area influences the relationship between the tactile sensation produced by stimulation of the reflex area and cortical activation in SI.

**Figure 1:**
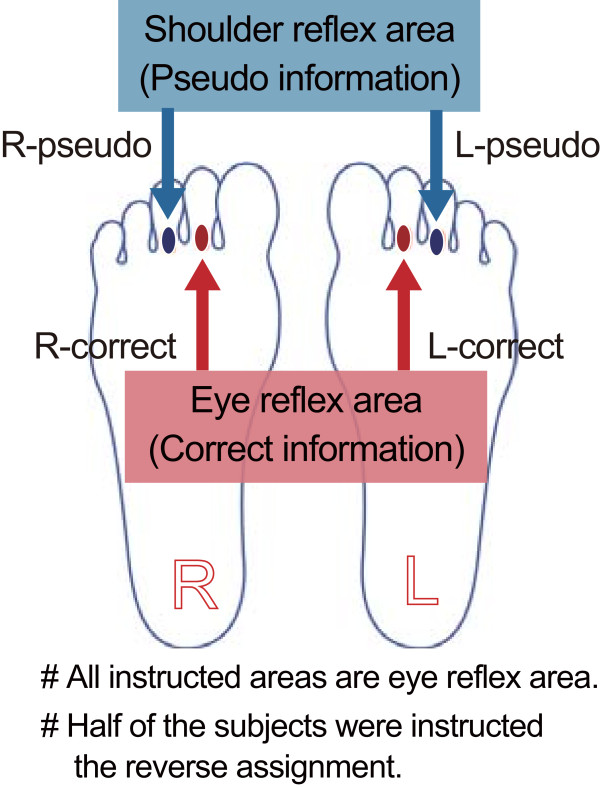
**Reflex area stimulated in the fMRI experiment. **Detailed legend: Location of the eye reflex area on the base of the second and third toes and assignment of the correct- and pseudo-reflex-area information for each reflex area.

In the present study, we used fMRI to investigate whether SI activation evoked by reflexological stimulation of a reflex area is affected by *a priori* information about the reflex area. We hypothesized that if stimulation of the reflex area activated SI representation of the body part corresponding to the reflex area regardless of the accuracy of the *a priori* information, then the somatotopical relationship between stimulation of the reflex area and neural activity in SI was robust. The eye reflex area was chosen as a stimulation point because it induced significant activation of the face representation area in the SI in our previous study [14]. The eye reflex area is located in two places: at the base of the second and third toes. Thus, we were able to test the hypothesis in a reflex area corresponding to the same body part by assigning correct (eye reflex area) or pseudo- (shoulder reflex area) information to separate toes (Figure 1). Either the correct or the pseudo-information was given to participants just before the fMRI was performed.

Additionally, we examined whether laterality of the foot reflex area was consist with the expected projection to SI. Our previous study [14] showed significant activation of the face representation area in the left SI during stimulation of the eye reflex area on the right foot. However, the lateral relationship between stimulation of the reflex area on the contralateral foot and cortical activation in SI has not been investigated. Thus, the present fMRI experiment had four experimental conditions: participants were stimulated at the two eye reflex areas on the right and left foot with each toe associated with either the correct- or pseudo-information condition. A two-factor factorial analysis of variance (ANOVA) was performed to clarify the effect of the accuracy of the assigned reflex-area information and the laterality of the cortical activation of the stimulated reflex area in SI.

## Methods

### Participants

Thirty-two healthy Japanese volunteers participated in the present study (21 males and 11 females; mean age, 22 ± 2 years, range, 20–31 years). All participants were judged to be right-handed according to the Edinburgh handedness inventory [18]. They had no history of neurological or psychiatric disease. It was confirmed that no participant had detailed knowledge of reflexology. All participants provided written informed consent, and the experimental protocol was approved by the Ethical Committee of the Tohoku University School of Medicine. The experiments were undertaken in compliance with national legislation and the Code of Ethical Principles for Medical Research Involving Human Subjects of the World Medical Association (Declaration of Helsinki).

### Experimental procedure

The present experiment was a double-blind study. Before the fMRI scan, each subject was acquainted with the reflex areas on the base of the second and third toe of their feet by an instructor. Half of the subjects were told that the base of their second toe was the eye reflex area and that the base of their third toe was the shoulder reflex area. Thus, those subjects received correct information about the reflex area for the base of the second toe and pseudo-information about the reflex area for the base of the third toe on both feet. The other half of the subjects received the opposite instructions, i.e., they received pseudo-information for the second toe and correct information for the third toe on both feet. These instructions are summarized in Figure 1. After providing this information, the instructor marked each reflex area on the subject’s feet. An experimenter and an assistant (who were not the instructor) entered the MRI scanner room together with the subject. The subject was placed in a supine position in the MRI scanner. To prevent head movements, the subject’s head was fastened by a band, and the space between the head coil and the subject’s head was stuffed with sponge pads. A semi-lucent screen was put in front of the subject’s face, and the subject was instructed to gaze at the fixation cross on the screen throughout the fMRI measurement. The experimenter wore a pair of headphones to receive an auditory cue, which was presented using Presentation software (Neurobehavioral Systems, Albany, CA, USA). The experimenter stimulated each reflex area using a wooden stick with the right hand in accordance with the auditory cue. Because this was a double-blind experiment, the experimenter and assistant did not know whether the correct or the pseudo-information was associated with each reflex area. The strength of pressure stimulation was adjusted according to each subject’s self-assessment immediately prior to the fMRI scan. The experimenter stimulated each reflex area on the subject in the MRI, and the stimulation strength was adjusted by ascertaining a comfortable level for each subject. The assistant held the subject’s feet throughout the scanning procedure.

The fMRI experiment was conducted using a block design consisting of four sensory stimulation tasks that involved stimulating the base of the second or third toes of subjects’ feet. The duration of each block was 5 s, with a 10-s interval between blocks. The experimenter continuously stimulated one reflex area in each task block. Each task was repeated 10 times; a 26-s rest period was imposed before the first block, and a 24-s rest period was imposed after the last block. Thus, the total time of fMRI scanning was 10 min 50 s. The order of tasks was counterbalanced among the subjects.

### fMRI data acquisition

All images were acquired using a Philips Achieva 3T MRI scanner (Philips, Amsterdam, The Netherlands). The fMRI time series data covering the entire brain was acquired using a T2-weighted gradient echo echo-planar imaging (GE-EPI). The parameters of the experiment were as follows: repetition time (TR), 2,500 ms; echo time (TE), 30 ms; flip angle (FA), 80° 42 slices; field of view (FoV), 192 × 192 mm; 64 × 64 matrix; slice thickness, 3 mm; slice gap, 0 mm. We acquired 260 scans per subject. To acquire a fine structural whole-head image, magnetization-prepared rapid-acquisition gradient-echo (MP-RAGE) images were obtained (TR, 6.5 ms; TE, 3 ms; FA, 8° FoV, 240 × 240 mm; 240 × 240 matrix; 162 slices; slice thickness, 1.0 mm).

### Data analysis

Data preprocessing and statistical analyses of the fMRI data were carried out using statistical parametric mapping software (SPM8; Wellcome Trust Center for Neuroimaging, London, UK). The effect of head motion across the scans was corrected by realigning all images to the first one. No data were excluded from subsequent analyses based on our head-motion exclusion criteria (i.e., head motion in the fMRI scanner greater than 3 mm). The structural image volume was then co-registered with the mean image of realigned echo-planar image (EPI). All EPI were spatially normalized to the Montreal Neurological Institute (MNI)-T1 template using the parameter to co-register and to normalize the structural image for the MNI-T1 template obtained by a segmentation process for each subject. Finally, each scan was smoothed with a Gaussian filter in a spatial domain (8-mm full-width at half maximum).

The fMRI data were analyzed using a two-stage approach in SPM8. In the first-level analysis, the hemodynamic responses produced by different experimental conditions were assessed at each voxel using a general linear model on an intrasubject basis. The analysis was based on the hypothesis that the hemodynamic responses were induced by the stimulation of the eye reflex area on the right foot with correct information [R(correct)] and pseudo [R(pseudo)] and the stimulation of the eye reflex area on the left foot with correct information [L(correct)] and with pseudo-information [L(pseudo)]. The hemodynamic response was assumed to be the canonical hemodynamic response function of 5-s duration. Global intensity normalization was performed on the average within-brain fMRI signal in each image, and low-frequency confounding effects were removed using a high-pass filter with a 128-s cutoff. A multiple regression analysis was performed on each voxel to detect the regions where MR signal changes were correlated with the hypothesized model to obtain the partial regression coefficients of each voxel.

The second-level analysis was performed using a two-way repeated-measures ANOVA on an intersubject basis. One factor was the laterality of the stimulated reflex area (left or right; two conditions), and the other factor was the accuracy of the assigned reflex-area information (correct or pseudo; two conditions). Furthermore, we performed a subtraction analysis of the main effect, which showed significant differences and both common and respective activation in the four experimental conditions. To depict an activation peak in the SI region, an anatomical mask image of the bilateral postcentral gyrus was used as an inclusive mask in the results section of SPM together with the whole-brain analysis. The mask image was generated using the SPM Anatomy toolbox [19]. The statistical threshold was set at *p* < 0.05 (corrected for family-wise error (FWE) by voxel level). Additionally, to assess the potential influence of sex differences, a three-way repeated-measures ANOVA with the factors stimulated reflex area laterality, accuracy of the assigned reflex-area information, and sex (male or female; two conditions) was performed. The main effect of sex and the interaction with other factors were tested with a statistical threshold of *p* < 0.05, corrected for FWE.

To examine activation induced under each experimental condition on an individual level, we created plots of the average activity levels within the regions of interest (ROIs) across subjects to evaluate local signal changes in the activated regions. This analysis was carried out using the MarsBaR toolbox [20]. ROIs were 4-mm-radius spheres centered within activation foci obtained by the second-level analysis. Because we focused on SI activity, an anatomical mask image of the bilateral postcentral gyrus was used to extract activation foci from the statistical analysis. The mask image was the same image used in the second-level analysis. Additionally, a portion of shoulder representation in SI [× = 34, y = -48, z = 70] that showed significant activation when the shoulder reflex area was stimulated in our previous study [14] and its contralateral side [× = -34, y = -48, z = 70] were used as ROIs to determine whether the pseudo-information stimulated the shoulder reflex area. A time series of percent signal changes in each ROI per scan from the beginning of each experimental condition (5 s) to the next resting condition (10 s) was extracted from preprocessed fMRI data on each subject, and the average time series of percent signal change across subjects was calculated.

## Results

**Figure 2:**
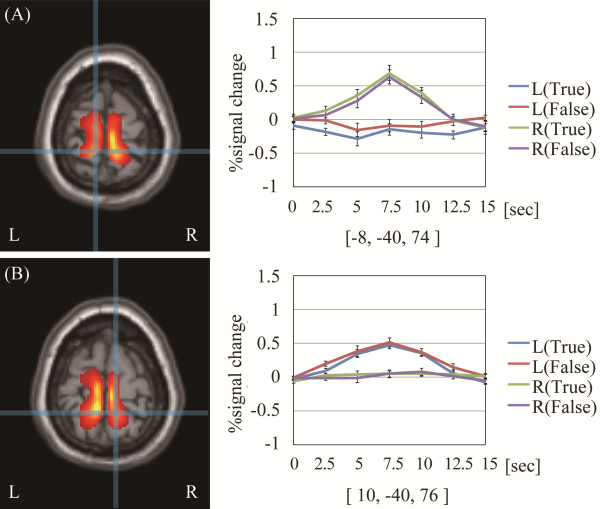
**Cortical areas showing a significant main effect of reflex-area laterality in SI and its activation plot. **Detailed legend: Results of second-level analysis showing a significant main effect of reflex area laterality [*F*-contrast of {L(correct) + L(pseudo)} versus {R(correct) + R(pseudo)}] superimposed on axial sections of the MNI single-subject template. The crosshairs show the activation focus peak position on the (**A**) left and (**B**) right paracentral lobules. Each plot shows an average time series of percent signal change across the subjects on the (**A**) left and (**B**) right paracentral lobules. The horizontal axis represents time elapsed from the onset of each condition. The error bars show the standard error of the mean.

The results of the second-level two-way repeated measures ANOVA are summarized in Table 1. The main effect of reflex-area laterality showed a significant bilateral difference in a broad region that included the superior SI, where the somatotopic representation of the foot is generally found, and the adjacent regions of the precentral gyrus (BA 4a) and superior parietal lobule. Additionally, the bilateral medial prefrontal region, insula, and cerebellum also showed significant differences. In contrast, no significant difference was found for the main effect of correct/pseudo-information or the interaction between information and laterality. Figure 2 shows the cortical regions where a significant difference in the main effect of laterality was observed in superior SI and an average time series of the percent signal changes of subjects for each SI activation peak at the onset of each experimental condition. The cortical areas significantly activated in each subtraction analysis are shown in Table 2. From the direct comparison of the left-foot versus the right-foot stimulation, significant activations were observed in the superior part of SI and in the neighboring motor or parietal region and insula in the stimulated foot’s contralateral hemisphere and in the ipsilateral cerebellar anterior lobe (Lobule V). Conversely, subtraction analysis between reflexological stimulation and true versus pseudo-information did not reveal any significant activation. The bilateral portions of the shoulder representation in SI were not significantly activated by stimulation with pseudo-information even though the statistical threshold was set at a more liberal threshold (*p* < 0.05, uncorrected).

**Table 1:** Cortical areas showing significant differences in the two-way repeated-measures ANOVA

**Area**	**Brodomann’s area**	**MNI coordinate**			**F-score**
		**x**	**y**	**z**	
Main effect of truth of assigned reflex area information					
N.S.					
Main effect of laterality of reflex area					
L. middle cingulate gyrus	BA6	-8	-4	46	61.69
R. supplementary motor area	BA6	8	6	48	60.58
L. paracentral lobule	BA4a	-6	-38	66	374.94
	**BA3b**	**-8**	**-40**	**74**	**184.84**
R. paracentral lobule	BA4a	6	-28	72	276.24
	**BA3b**	**10**	**-44**	**70**	**173.63**
L. insula		-34	-24	18	42.42
R. insula		32	-24	18	37.39
L. Cerebellum (lobule V)		-14	-40	-24	125.91
R. Cerebellum (lobule V)		14	-42	-24	156.89
Interaction between two factors					
N.S.					

The activation peak in **bold **was observed within SI area defined by the Anatomy toolbox [20].

**Table 2:** Cortical areas showing significant activation in each subtraction analysis

**Area**	**Brodomann’s area**	**MNI coordinate**			**T-score**
		**x**	**y**	**z**	
Stimulation of reflex area on left foot: {L(correct) + L(pseudo)} – {R(correct) + R(pseudo)}					
R. supplementary motor area	BA6	8	-6	48	7.78
R. paracentral lobule	BA4/6a	6	-28	72	16.62
	**BA3b/4a**	**8**	**-40**	**74**	**15.32**
R. rolandic operculum		44	-32	22	5.10
R. insula		32	-24	18	6.11
R. thalamus		16	-24	0	5.08
L. cerebellum (lobule V)		-14	-40	-24	11.22
Stimulation of reflex area on right foot: {R(correct) + R(pseudo)} – {L(correct) + L(pseudo)}					
L. middle cingulate gyrus	BA6	-8	-4	46	7.85
L. paracentral lobule	BA4a/6	-6	-38	66	19.36
	BA4	-6	-26	70	15.37
	**BA3b/4a**	**-10**	**-44**	**70**	**14.07**
L. insula		-34	-24	18	6.51
R. cerebellum (lobule V)		14	-42	-24	12.53
Stimulation of reflex area with true information: {L(correct) + R(correct)} – {L(pseudo) + R(pseudo)}					
N.S.					
Stimulation of reflex area with pseudo information: {L(pseudo) + R(pseudo)} – {L(correct) + R(correct)}					
N.S.					

The activation peak in **bold **was observed within SI area defined by the Anatomy toolbox [20].

**Figure 3:**
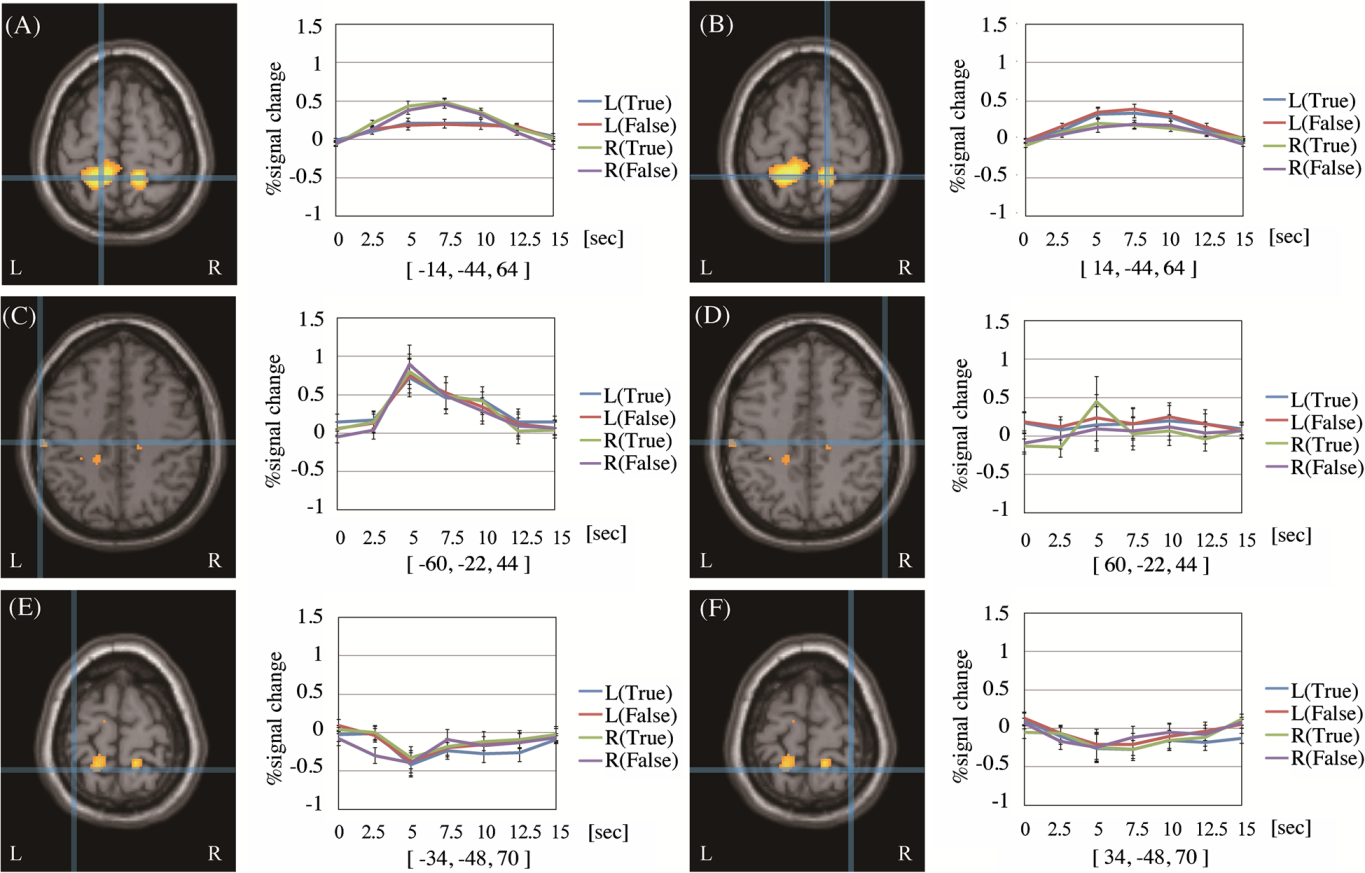
**Cortical areas showing common activation foci under the four conditions and activation plots. **Detailed legend: Results of second-level analysis showing common activation foci under the four conditions [L(correct) + L(pseudo) + R(correct) + R(pseudo)] superimposed on axial sections of the MNI single-subject template. Crosshairs show the activation focus peak position on the (**A**) left and (**B**) right paracentral lobules and the (**C**) left middle part of the postcentral gyrus. Crosshairs at (**D**), (**E**), and (**F**) show the center point of each designated ROI. The statistical threshold was set at *p* < 0.05, corrected for family-wise error. Plots show an average time series of percent signal changes across subjects on the (**A**) left and (**B**) right paracentral lobules and the (**C**) left middle part of the postcentral gyrus. Plot (**D**) shows an average time series of percent signal change across subjects on the contralateral coordination (**C**). Plots (**E**) and (**F**) indicate each average time series of percent signal changes across subjects on the bilateral shoulder representation in SI determined by the location obtained from stimulation of the shoulder reflex area in our previous study [14] and its contralateral coordination. The horizontal axis represents time elapsed from the onset of each condition. The error bars show the standard error of the mean.

The cortical areas that showed significant common activation across the four conditions are summarized in Table 3, and the activation peaks within the SI region obtained by each of the four experimental conditions are summarized in Table 4. Reflexological stimulation of each foot reflex area activated the superior part of the contralateral SI hemisphere. Stimulation of both eye reflex areas significantly activated the left middle part of the postcentral gyrus, where the somatotopic representation of the face is generally found. However, the homologous contralateral region of the middle part of the postcentral gyrus was not significantly activated by stimulation of the eye reflex areas even though the statistical threshold was set at more liberal threshold (*p* < 0.05, uncorrected). Moreover, we observed significant bilateral activation of the paracentral lobule, located slightly lateral to the area where the main effect of reflex area laterality was located, and observed that the activation focus was generally the somatotopic representation of the lower limb. Moreover, significant activation in the bilateral motor region, secondary somatosensory area (SII) within the rolandic operculum, insula, and thalamus was observed. Figure 3 shows the common activation peak in the SI area observed under all conditions and an average time series of the percent signal changes in each activated area and additional ROIs: the contralateral homologous region of the left middle part of the postcentral gyrus and the bilateral shoulder representation in SI determined by the location that showed increased activity in response to stimulation of the shoulder reflex area in our previous study [14]. A stimulation-related signal change was also observed in the averaged individual time series data in the bilateral paracentral lobule and left middle SI, whereas the right contralateral portion of the middle SI and expected SI shoulder representation did not show any significant signal changes.

**Table 3:** Cortical areas showing common activation under four experimental conditions

**Area**	**Brodomann’s area**	**MNI coordinate**			**T-score**
		**x**	**y**	**z**	
Common activation to four conditions: L(correct) + L(pseudo) + R(correct) + R(pseudo)					
L middle cingulate gurus		-14	-32	40	5.38
L posterior cingulate gurus		-8	-44	20	5.98
**L. postcentral gyrus**	**BA3b**	**-14**	**-44**	**64**	**7.60**
	**BA1**	**-60**	**-22**	**44**	**5.09**
**R. postcentral gyrus**	**BA3b**	**14**	**-44**	**64**	**6.76**
L. rolandic operculum		-48	-22	16	8.53
		-54	-2	8	7.99
R. rolandic operculum		54	0	8	7.19
		48	-26	20	7.02
L. insula		-38	0	12	9.39
		-34	-20	16	9.57
R. insula		38	8	12	9.59
		34	-22	14	9.57
L. thalamus		-8	-10	6	7.67
R. thalamus		18	-12	10	8.39
L. Amygdala		-20	-2	-6	5.33
R. Amygdala		20	-4	-6	5.10

The activation peak in **bold **was observed within SI area defined by the Anatomy toolbox [20].

**Table 4:** Comparison of SI activation peaks observed under four experimental conditions

**Area**	**Condition**	**MNI coordinate**			**T-score**
		**x**	**y**	**z**	
Left superior part of SI area					
	L(True)	-18	-42	60	5.21
	L(pseudo)	-18	-42	60	5.49
	R(True)	-12	-48	71	9.88
	R(Pseudo)	-12	-44	66	9.50
Right superior part of SI area					
	L(True)	14	-42	70	8.03
	L(pseudo)	12	-44	68	8.22
	R(True)	16	-44	60	4.98
	R(Pseudo)	16	-40	65	5.24
Left middle part of SI area					
	*L(True)*	*-60*	*-22*	*44*	*4.27*
	L(pseudo)	-58	-24	48	4.87
	R(True)	-58	-22	44	4.82
	R(Pseudo)	-60	-26	49	4.96

The SI area mask image was created using the Anatomy toolbox [20]. The statistical threshold was set at *p* < 0.05, corrected for FWE. The activation peak in *italics *was observed under a liberal threshold (*p* < 0.001, uncorrected).

The results of the second-level three-way repeated-measures ANOVA, which assessed the influence of sex differences, revealed no significant differences for the main effect of sex, in the two-way interaction between sex and correct/pseudo-information and between sex and laterality, or in the three-way interaction among sex, correct/pseudo-information, and laterality.

## Discussion

The present study aimed to test whether SI neural activity induced by tactile stimulation at reflexological stimulation sites was affected by *a priori* knowledge about reflexology. The results suggest that a robust relationship exists between tactile stimulation of the eye reflex area and neural activity representing the somatosensory percept of the reflexological stimulation of the corresponding body part. A second-level ANOVA revealed no significant effect of pseudo-information about the reflex area. The middle part of the postcentral gyrus, which is associated with face representation in SI [21-23], showed significant common activation by all of the eye reflex areas. Thus, neural activity corresponding to the perception of reflexological stimulation was not affected by *a priori* knowledge about the reflex area. These results support our previous finding that reflexological stimulation induces a somatosensory process corresponding to the stimulated reflex area [14]. Several neuroimaging studies of acupuncture have also suggested that stimulation of different acupoints on upper and lower limbs led to activation in somatotopically different locations [8,24]. However, that relationship basically showed a connection between the acupoint location on a body part and the SI somatotopic representation; however, an acupoint does not necessarily have a clear relationship with a specific body part. Laser stimulation of a specific acupoint empirically related to ophthalmic disorders reportedly induces visual association cortex activation [25]. Therefore, these differential findings may imply that different neural mechanisms are involved in processing perceptions of reflexological and acupunctural stimulation.

Furthermore, our results indicate that the reflexological stimulation of the eye reflex area induces left-lateralized activation in the middle part of the postcentral gyrus, as the contralateral side (right) was not significantly activated. Anatomically, the final projection of face tactile sensation is to the middle part of the postcentral gyrus of the contralateral hemisphere via the trigeminal nerve. Neuroimaging studies have shown functional lateralization of face representation in SI. For example, Eickhoff et al. [21] reported that unilateral stimulation of the face induced bilateral activation of the middle part of SI. Another fMRI study demonstrated that the lateral relationship between tactile stimulation and SI activity differed depending on which part of the face is stimulated [22], and Moulton et al. [23] suggested that face representation in SI is arranged segmentally by subdivision of the trigeminal nerve. These discrepancies may be explained by the present results, which indicate that the somatotopic relationship between the reflexological stimulation of the eye reflex area and the neural processing of tactile sensations emanating from the corresponding body part is characterized by a lateral pattern that differs from that of the actual relationship and that the stimulation of the eye reflex area was perceived as a tactile sensation emanating from a more specific part of the face. Two possible interpretations of the left-lateralized SI activation can be offered. First, it may be that all of the eye reflex areas we stimulated correspond to a unilateral face representation and that the reflex area corresponding to the contralateral eye is expressed in another area. Alternatively, tactile sensations activated by reflexological stimulation of the eye reflex area may be processed primarily in the left hemisphere of SI. It is necessary to examine the relationship between SI activity and the stimulation of reflex areas corresponding to other body parts to determine whether either of these interpretations is valid. Balslev et al. [26] reported that cortical activation-related eye muscle proprioception was located at a slightly anterior portion of the middle sensorimotor cortex area compared with our results (x, y, z = -36, -16, 40 for right eye and x, y, z = -38, -12, 42 for left eye, on left sensorimotor cortex) and that the activation peak specifically for eye movement was located in a more anterior location. The present study did not investigate the relationship between reflexological stimulation and induced muscle activation on the corresponding body part because we did not measure related muscle activation, such as eye movement, using electromyography or eye tracking. However, specific cortical activation-related eye movement, such as in the frontal eye field, was not observed in the present results. In contrast with the results reported by Balslev et al. [26], we observed that activation of the middle SI might be reflected in activity induced not by voluntary eye movement but by the perception of stimulation.

Activation of another SI location that corresponded to the stimulation of each foot was observed. Stimulation of the eye reflex area on each foot induced significant activation in the contralateral hemisphere of the primary sensorimotor cortex, medial prefrontal region, SII, insula, and the ipsilateral hemisphere of the anterior part of the cerebellum (lobule V). This relationship between somatosensory stimulation and laterality was already suggested in a previous study of somatosensory processing [27]. Tactile sensations of the foot project to the superior part of SI [28], and our previous study demonstrated a consistent relationship between reflexological stimulation of the left foot and cortical activation in the right superior part of SI [14]. Furthermore, the activated region spread to neighboring areas, including the primary motor area (area 4a/6) and superior parietal lobule. Yoo et al. [10] reported that real acupunctural stimulation induced broader activation in the sensorimotor cortex and posterior parietal region and area 3a was selectively activated by real acupunctural stimulation as compared with sham acupunctural stimulation. Although the present result reflected a similar trend in the activation pattern, only a few overlapping areas were observed on a probabilistic map of area 3a, as defined by the Anatomy toolbox. This difference might be due to a difference in the stimulation procedure used by reflexology and acupuncture. As area 3a is responsive to proprioceptive sensation and reportedly responds to kinesthetic and motor stimulation [29], foot kinesthetic responses induced by reflexological stimulation might differ from those induced by acupunctural stimulation. The activation foci located in a more lateral portion of the superior SI were observed during bilateral stimulation of both feet, although the activation plot showed that the activity on the contralateral side tended to be greater than that on the ipsilateral side. In terms of the somatotopic representation of the foot in SI, the activation focus becomes more lateral when the more anterior portion of the leg is stimulated [30,31]. In our experiment, the assistant held the subjects’ feet in the same position during fMRI measurement. When the reflex area was stimulated, the assistant should have kept the subjects’ feet in the same position as in the resting state; indeed, the activity in this area reflected the tactile sensation that arose from foot-position fixation.

Our results showed a robust somatotopic relationship between stimulation of the eye reflex area and activation of the middle part of SI, with no effect of *a priori* information regarding the reflex area. However, our study had several limitations. We were unable to specify the exact relationship between a tactile sensation of the reflex area and a specific part of the face. We were also unable to elucidate the mechanism underlying the beneficial effect of reflexological treatment because our experimental setup was focused on the relationship between SI cortical activation and erroneous reflexology information. Further, the present study did not clarify the relationship between reflexological stimulation and induced muscle activation on eye or shoulder movement because we did not measure muscle activation using electromyography or eye tracking. As for the integrated study of neuroscience and acupuncture, we clarified a metabolic contributor to the local antinociceptive effect of acupuncture [6]. Reflexological stimulation induces changes in resting-state neural activity [13], and it is possible that this kind of change in resting-state neural activity might be a potential confounder of our experimental results. Thus, a multifaceted, systematic investigation that includes neuroimaging approaches is needed to reveal the physiological basis of reflexology.

## Conclusions

The relationship between cortical activity and reflexological stimulation is robust, even when *a priori* pseudo-information about the reflex area is given to subjects. The results of the present study support our previous finding showing that reflexological stimulation of the eye reflex area induced significant activity in the face representation area in SI of the left hemisphere and in the foot representation area in SI of the contralateral hemisphere.

## Abbreviations

fMRI: Functional magnetic resonance imaging; SI: Primary somatosensory cortex; SII: Secondary somatosensory cortex; EEG: Electroencephalography; ANOVA: Analysis of variance; GE-EPI: Gradient echo echo-planar imaging; TR: Repetition time; TE: Echo time; FA: Flip angle; FoV: Field of view; MP-RAGE: Magnetization-prepared rapid-acquisition gradient-echo; EPI: Echo-planar image; MNI: Montreal neurological institute; FWE: Family-wise error; ROI: Region of interest.

## Competing interests

The authors declare that they have no competing interests.

## Authors’ contributions

NM participated in the design of the study, performed the fMRI experiment and statistical analysis, and drafted the manuscript. YA participated in the design of the study, performed the fMRI experiment, and helped to draft the manuscript. AS participated in the design of the study, performed the fMRI experiment, and helped to draft the manuscript. RK participated in the design and coordination of the study and helped to draft the manuscript. All authors read and approved the final manuscript.

## Pre-publication history

The pre-publication history for this paper can be accessed here:

http://www.biomedcentral.com/1472-6882/13/114/prepub
